# Origins of Metabolic Pathology in *Francisella*-Infected *Drosophila*

**DOI:** 10.3389/fimmu.2020.01419

**Published:** 2020-07-08

**Authors:** Crystal M. Vincent, Carolina J. Simoes da Silva, Ashima Wadhawan, Marc S. Dionne

**Affiliations:** MRC Centre for Molecular Bacteriology and Infection and Department of Life Sciences, Imperial College London, London, United Kingdom

**Keywords:** *Drosophila*, *Francisella*, metabolism, pathophysiology, immune response

## Abstract

The origins and causes of infection pathologies are often not understood. Despite this, the study of infection and immunity relies heavily on the ability to discern between potential sources of pathology. Work in the fruit fly has supported the assumption that mortality resulting from bacterial invasion is largely due to direct host-pathogen interactions, as lower pathogen loads are often associated with reduced pathology, and bacterial load upon death is predictable. However, the mechanisms through which these interactions bring about host death are complex. Here we show that infection with the bacterium *Francisella novicida* leads to metabolic dysregulation and, using treatment with a bacteriostatic antibiotic, we show that this pathology is the result of direct interaction between host and pathogen. We show that mutants of the immune deficiency immune pathway fail to exhibit similar metabolic dysregulation, supporting the idea that the reallocation of resources for immune-related activities contributes to metabolic dysregulation. Targeted investigation into the cross-talk between immune and metabolic pathways has the potential to illuminate some of this interaction.

## Introduction

Infection phenotypes can result from direct interactions between host and pathogen, or indirect interactions which often take the form of trade-offs with, or damage caused by, the host's immune response (1–4). The deleterious effects of immune activity are most often described in the context of an unfettered immune response and the resultant “costs” of said activation [e.g., decreased lifespan (5–7)], or, the by-products of immune effectors [e.g., biochemical interactions; pleiotropic signaling cascades (8–11)].

While the contribution of the host's immune response to infection pathology is acknowledged, a majority of studies focus on pathogen-derived infection phenotypes. Studying direct host-pathogen interactions allows researchers to be on the frontline of infection, dissecting each play and counter-play as host and pathogen battle, often resulting in either elimination of the invader, or death of the host. Work in the fruit fly has supported the assumption that mortality resulting from bacterial invasion is largely due to direct host-pathogen interactions, as lower pathogen loads are often associated with reduced pathology, and bacterial load upon death is predictable (12–14).

*Drosophila melanogaster* has been used as a model for host-pathogen interactions due to its experimental tractability (10, 15–18). One emergent and exciting field of work is focused on the interaction between infection and host metabolism. Work from our lab and others has identified a number of regulatory factors that play roles in both immune and metabolic activity (10, 11, 19). For example, infection with the intracellular bacterium *Mycobacterium marinum* leads to a wasting phenotype, with flies losing both glycogen and triglyceride stores over the course of infection (20). This wasting phenotype is mediated, in part, by impaired insulin signaling.

Here we explore the interaction between pathogenesis, immunity and metabolic function. The bacteriostatic antibiotic doxycycline, a close analog of tetracycline, is a standard treatment for infection with *Francisella tularensis* (21). Flies infected with the Gram-negative intracellular bacterium *Francisella novicida* die within 6 days of infection (22). In these infections, the greatest pathogen loads are observed immediately prior to death (22). We show that *F. novicida—*infected flies treated with tetracycline maintain live bacteria at loads less than the initial inoculum and exhibit no difference in survival with their PBS-injected controls. These results suggest that factors dependent on bacterial proliferation contribute to mortality during infection. We find that the metabolic dysregulation observed during this infection is dependent on bacterial load, and we distinguish between immune-derived and pathogen-driven pathology using immune deficient hosts.

## Materials and Methods

### General Experimental Procedures

We used *w*^1118^; and *w*^1118^; *imd*^10191^ flies in this study. The *w*^1118^; *imd*^10191^ line is a mutant of the immune deficiency pathway (*imd*); it has a 26-nucleotide deletion that frameshifts the protein at amino acid 179, which is the beginning of the death domain (23). Male flies were collected following eclosion and kept in same-sex vials for 5 to 9 days in groups of 20. Flies were maintained on a standard sugar-yeast diet (10% yeast, 8% fructose, 2% polenta, 0.8% agar, supplemented with 0.75% propionic acid and 0.075% nipagin) at 25°C. Injections were carried out using a pulled-glass capillary needle and a Picospritzer injector system. Flies given tetracycline were transferred to 0.04% tetracycline food (same recipe as above, supplemented with powdered Tetracycline≥98.0% (NT), 87128 Sigma-Aldrich) 6 h after injection. We transferred flies onto tetracycline 6 h post injection for two reasons: first, we wanted to simulate normal usage of antibiotics, and therefore did not administer tetracycline prior to the establishment of infection; second, preliminary studies showed that flies transferred to tetracycline food at 6 and 24 h post infection had similar survival (Supplementary Figure 1). We chose to administer tetracycline at 6 h to allow more time to assay pathology in the shorter-lived *imd*^10191^ mutants.

Bacteria were grown from single colonies overnight at 37°C in a shaking incubator. Wild-type *Francisella novicida* (U112) and tetracycline resistant *Francisella novicida* (U112 pKK219-GFP) were grown in Tryptic Soy Broth supplemented with 5% cysteine. U112 pKK219 cultures were additionally supplemented with 0.1 % tetracycline. Each fly was injected with 50 nl of bacterial culture diluted to OD_600_ = 0.1 in PBS. As infection (bacteria) and wounding controls, we had flies that were injected with sterile PBS and anesthetized but otherwise unmanipulated, respectively.

### Survival Assays

Survival experiments were performed at 29°C with 15–25 flies/vial. Survival was monitored daily and flies were “tipped” into fresh vials every 3 days; this method for transferring flies permits the provisioning of fresh food without anesthesia.

### Bacterial Quantification

For each sample, at the timepoint specified, 1 fly was homogenized in a 100 μl of Tris-EDTA, 1% Proteinase K (NEB, P8107S) solution. Homogenates were incubated for 3 h at 55°C followed by a ten-minute incubation at 95°C. Following incubation, we performed our qPCR protocol as outlined below to determine the number of bacterial colony forming units (CFU). Methods used to estimate bacterial load upon death (BLUD) are described elsewhere (12); briefly, we collected flies within 30 min of death and processed samples as above. Preliminary experiments showed that quantification via qPCR yields similar results to plating (mean CFU: plating−1850, qPCR−2204, Supplementary Figure 2). As such, we are confident that this method of quantification provides a good estimate of bacterial number.

### Gene Expression—Quantitative Reverse Transcription PCR

For each sample, three flies were homogenized in 100 μl of the single-step RNA isolation reagent TRI Reagent (Sigma), followed by a chloroform extraction and precipitation in isopropanol. The resultant pellet was then washed with 70% ethanol. Pellets were resuspended and subjected to DNase treatment. Revertaid M-MuLV reverse transcriptase and random hexamers (Thermo Scientific) were used to carry out cDNA synthesis. Five-microliter of each cDNA sample was put into a “neat” standard tube; this tube was later used to generate standards which would be used to generate a standard curve for each gene. Each cDNA sample was diluted and this diluted sample used for analysis.

We used qPCRBIO SyGreen Mix for qRT-PCR. The cycling conditions were as follows: Hold 95°C for 10 min, then 45 cycles of 95°C for 15 s, 59°C for 30 s, 72°C for 30 s, followed by a melting curve. To gain a general picture of AMP activity, we assayed a subset of antimicrobial peptides which we have found to be strongly induced during *F. novicida* infection (Table 1). Gene expression was calculated based on the standard curve generated during each run, normalized to the value of our housekeeping gene, *RPL1*. Samples from PBS and infected treatments were then divided by the mean value of their uninfected controls to generate expression values relative to uninfected flies. All gene expression experiments were repeated at least twice, with four or more biological replicates per experiment.

**Table 1:** Primer sequences used for qRT-PCR.

**Gene**	**Forward**	**Reverse**
*AttA*	5′- cacaatgtggtgggtcagg−3′	5′- ggcaccatgaccagcatt−3′
*Dpt*	5′- accgcagtacccactcaatc−3′	5′- cccaagtgctgtccatatcc−3′
*Dro*	5′- ccatcgaggatcacctgact−3′	5′- ctttaggcgggcagaatg−3′
*Mtk*	5′- tcttggagcgatttttctgg−3′	5′- tctgccagcactgatgtagc−3′
*Rpl1*	5′- tccaccttgaagaagggcta−3′	5′- ttgcggatctcctcagactt−3′
*U112_IgID*	5′- aggataagacctgtctgca−3′	5′- ggttaagcaccgcaagctat−3′

### Measurement of Glucose and Glycogen Levels

Each sample contained three flies that were homogenized in 75 μl of TE + 0.1% Triton X-100 (Sigma Aldrich), and stored at −80°C. Prior to the assay, samples were incubated for 5 min at 65°C. Following incubation, 10 μl from each sample was loaded into 3-wells of a 96-well plate. Each well was designated to serve as a measurement for either: control (10 μl sample + 190 μl H_2_0), glucose [10 μl sample + 190 μl glucose reagent (Sentinel Diagnostics)], or glycogen [10 μl sample + 190 μl glucose reagent + amyloglucosidase (Sigma Aldrich)]. A standard curve was generated by serially diluting a glucose sample of known concentration and adding 190 μl of glucose reagent to 10 μl of each standard. Standards were always run at the same time and in the same plate as samples. Plates were incubated for 1.5–2 h at 37°C following which the absorbance for each well at 492 nm was determined using a plate reader.

### Measurement of Triglyceride Levels

Triglycerides were measured using Thin Layer Chromatography (TLC) assays as described elsewhere (24). Briefly, each sample consisted of eight flies; flies were placed in microcentrifuge tubes and stored at −80°C until the time of analysis. To perform the TLC assay, samples were removed from the −80°C freezer and spun down (3 min at 13,000 rpm at 4°C) in 100 μl of a 3:1 (v/v) mix of chloroform and methanol. Flies were then homogenized and subjected to a further “quick spin.” Standards were generated using lard dissolved in the same chloroform: methanol solution. We loaded 2 μl of each standard and 20 μl of each sample onto a silica gel glass plate (Millipore). Plates were then placed into a chamber pre-loaded with solvent (a 4:1 (v/v) mix of hexane and ethyl ether) and left to run until the solvent reached a point 1 cm short of the edge of the plate. Plates were then removed from the chamber, allowed to dry, and stained with CAM solution (24). Plates were baked at 80°C for 15–25 min and imaged using a scanner. Triglyceride was quantified in Image J using the Gel Analysis tool.

### Statistical Analysis

All data were analyzed in R Studio with R version 3.5.1. Survival data were analyzed using pairwise comparisons Log-Rank tests. BLUD assay correlations were computed by Pearson's correlation. For all other assays, we first tested for normality of data which dictated whether an ANOVA, *t*-test, Kruskal—Wallis analysis of variance, or Wilcoxon test was used to calculate differences between treatments. When appropriate, we performed *post-hoc* Tukey or Dunn analyses to identify specific differences between treatments. All assays were repeated at least twice with the number of biological replicates as indicated.

## Results

### Effect of Tetracycline During *F. novicida* Infection on *w*^1118^ Flies

**Figure 1:**
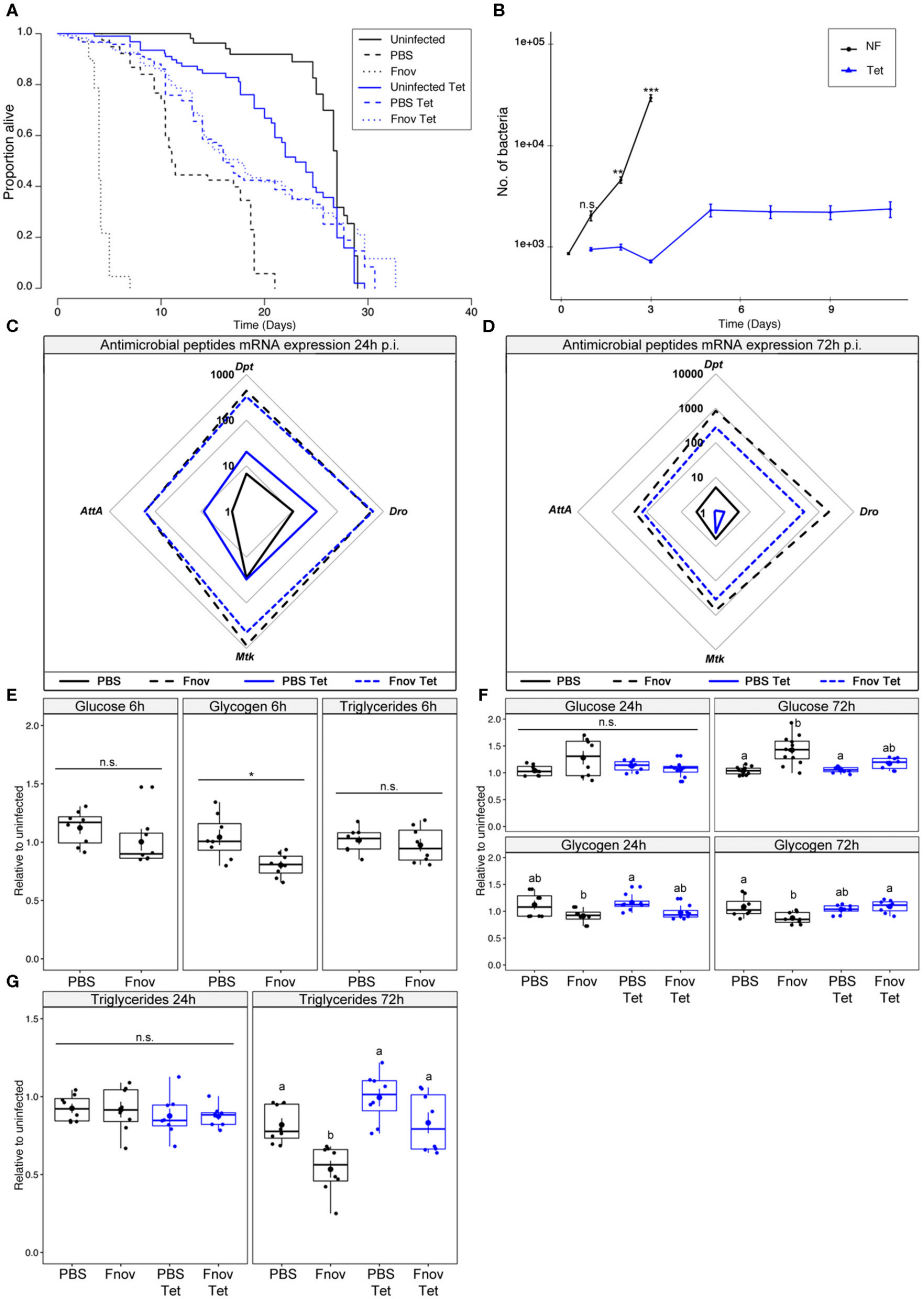
Tetracycline alleviates pathology associated with *F. novicida* infection. Five to nine days old adult *w*^1118^ flies infected with *F. novicida* (OD_600_ = 0.1, or ~1,000 bacteria). Animals fed tetracycline were switched to tetracycline food 6 h post-infection. In all plots, black and blue tracings represent normal and tetracycline food, respectively. Significance codes: *** <0.001, ** <0.01, * <0.05. Survival **(A)** *F. novicida* -infection is represented by dotted lines. Uninfected and PBS controls are represented by solid and dashed lines, respectively. Infected, tetracycline-fed flies live 4.3x longer than flies kept on normal food. Median survival of infected flies: tetracycline−17.5 d; normal food−4 d (Log-Rank = 745.6, df = 5, *n* = 602, *p* ≤ 2e-16). Survivals were repeated 2 or 3 times with 20 flies/treatment/repeat. A full-factorial report of statistics can be found in Supplementary Table 1. Bacterial quantification **(B)** tetracycline-fed flies (Tet) had significantly lower bacterial loads at both 2 and 3 d post infection (Kruskal-Wallis = 112.47, df = 11, *p* = 2.2e-16; Dunn's *post hoc*: 48 h–*p* = 0.001, 72 h–*p* = 2.1e-11). A significant increase in bacterial load was observed between days 3 and 5 in tetracycline-fed flies (Dunn's *post hoc*: tetracycline 2d—tetracycline 5 d, *p* = 0.008), but remained constant from that point on. Normal food (NF) flies show an exponential increase in bacterial load throughout infection. Markers indicate means and bars represent SE. Bacterial quantifications were repeated twice, with *n* = 8 samples/treatment/repeat. Spider plots showing antimicrobial peptide transcript levels 24 h **(C)** and 72 h **(D)** post infection. All tracings are relative to uninfected individuals of the same treatment. Solid and dashed lines represent PBS and *F. novicida* injection, respectively. The area contained within the innermost quadrilateral represents induction levels falling between one and ten times that of the uninfected controls. The middle and outer quadrilaterals represent 10–100 and 100–1,000-fold induction, respectively. Antimicrobial peptide assays were repeated twice, with four samples/treatment/repeat. These data are also shown, represented differently, in Supplementary Figure 3A. Metabolism **(E)** glucose, glycogen and triglyceride levels are unchanged at an early point (6 h post-infection) of infection. **(F)** Glucose and glycogen levels 24 and 72 h post-infection. Infection led to a significant increase in glucose levels during late infection in normal food flies (Kruskal-Wallis = 20.007, df =3, *p* = 1.7e-04; Dunn's *post hoc*: normal food PBS—normal food Fnov = 2.6e-04). Glycogen stores were depleted by infection in normal food flies (AOV: df =3, *n* = 28, *F* = 4.855, *p* = 7.6e-03, Tukey's HSD: normal food PBS—normal food Fnov = 0.015). Groups sharing the same letter are not significantly different. **(G)** Infection led to a significant depletion of triglycerides in infected normal food flies (AOV: df =3, *n* = 28, *F* = 11.86, *p* = 3.4e-05, Tukey's HSD: normal food PBS—normal food Fnov, *p* = 0.006). Infection did not affect any measure of metabolism in tetracycline-fed flies. Large circular markers indicate means while smaller circles represent individual data points. Horizontal bar within each box represents the median. The bottom and top lines of the box represent the 1st and 3rd quartiles, respectively. Whiskers represent either the maximum and minimum values, or, the maximum and minimum values falling within 1.5x the interquartile range, in which case outliers are indicated. Metabolic assays were repeated 2 or 3 times, with four samples/treatment/repeat.

Infected *w*^1118^ flies given tetracycline lived 4.3x longer than flies kept on normal food (median survival of infected flies: normal food−4 d; tetracycline−17.5 d; Figure 1A) and similar survival to their PBS controls (median survival of tetracycline-fed flies: PBS−16 d; *F. novicida*−17.5 d; Figure 1A, Supplementary Table 1). Flies injected with PBS and fed tetracycline lived significantly longer than their normal food controls, while the opposite was true in uninfected flies (median survival; Uninfected: normal food−27 d, tetracycline−23 d; PBS: normal food−11 d, tetracycline−16 d; Figure 1A). As tetracycline is a bacteriostatic antibiotic and thus prevents bacteria from proliferating rather than actively killing bacterial cells (21), we assayed bacterial load over the course of infection in tetracycline-fed and normal food flies. Confirming our preliminary findings, flies kept on normal food exhibited an exponential increase in bacterial numbers over the first 3 days of infection, while flies given tetracycline maintained a low level of bacteria (Figure 1B). Bacterial load differed significantly between normal food and tetracycline flies for all time points beyond 24 h. Furthermore, bacterial loads in normal food flies differed significantly between all consecutive time points apart from days 1 and 2. For tetracycline-fed flies, there was a significant increase in bacterial load between day 3 and day 5, but no difference between days 1 and 3, nor days 5 and 11 (Figure 1B).

Because pathogen detection is required for the initiation of the antimicrobial peptide (AMP) response (25–27), we sought to determine how the changing bacterial load on normal food, and the static load on tetracycline food, were affecting AMP expression. We found that 24 h post-infection, AMP expression did not differ between flies given tetracycline and those on normal food (Figure 1C, Supplementary Figure 3). This finding was unsurprising as bacterial loads did not differ significantly between the two groups at this time. In contrast, by 72 h post-infection, all four of the AMPs measured had greater induction in normal food flies compared to tetracycline-fed (Figure 1D).

Having confirmed that infection with *F. novicida* is lethal, and characterized by rapid bacterial proliferation and AMP induction, we wanted to determine if *F. novicida* infection had any effect on host metabolism. Infected tetracycline-fed flies live longer, have lower bacterial loads, and reduced AMP induction; thus, we anticipated that the metabolism of these flies would not be affected in the same way as flies that were not given tetracycline. However, we found that flies infected with the non-lethal pathogen *Microccocus luteus* exhibit metabolic dysregulation similar to what we here observe, suggesting that this phenotype is independent of moribundity [Supplementary Figure 4, (28)]. During the early stages of infection, there was no effect of tetracycline or infection on metabolism (Figures 1E,F). However, by 72 h post-infection, infected flies kept on normal food had significantly higher levels of glucose, as well as depleted glycogen and triglyceride stores. Infection did not affect glucose, glycogen, or triglyceride levels in tetracycline-fed flies (Figures 1F,G). Collectively, these data show that tetracycline reduces the pathology of *F. novicida* infection.

### Effect of Tetracycline During *F. novicida* Infection on *imd*^10191^ Flies

Mutants of the Imd pathway exhibit poor AMP induction in response to infection with Gram-negative bacteria and impaired survival during infection with *F. novicida* (22, 25, 29, 30), so we did not measure AMPs in these flies. Instead, we tested whether treatment with tetracycline enhances survival in *imd*^10191^ mutants to a level similar to that observed in *w*^1118^ flies. In addition, as there is an assumed “cost of immunity” (4, 31–33), infection of *imd*^10191^ mutants with *F. novicida* would allow us to determine if part of the observed metabolic dysregulation during infection in *w*^1118^ is caused by *imd*-dependent immune activation. Any observed differences in metabolism between *w*^1118^ and *imd*^10191^ flies are unlikely to result from differential resource availability as we found no difference in triglyceride levels between the two (Supplementary Figure 5).

**Figure 2:**
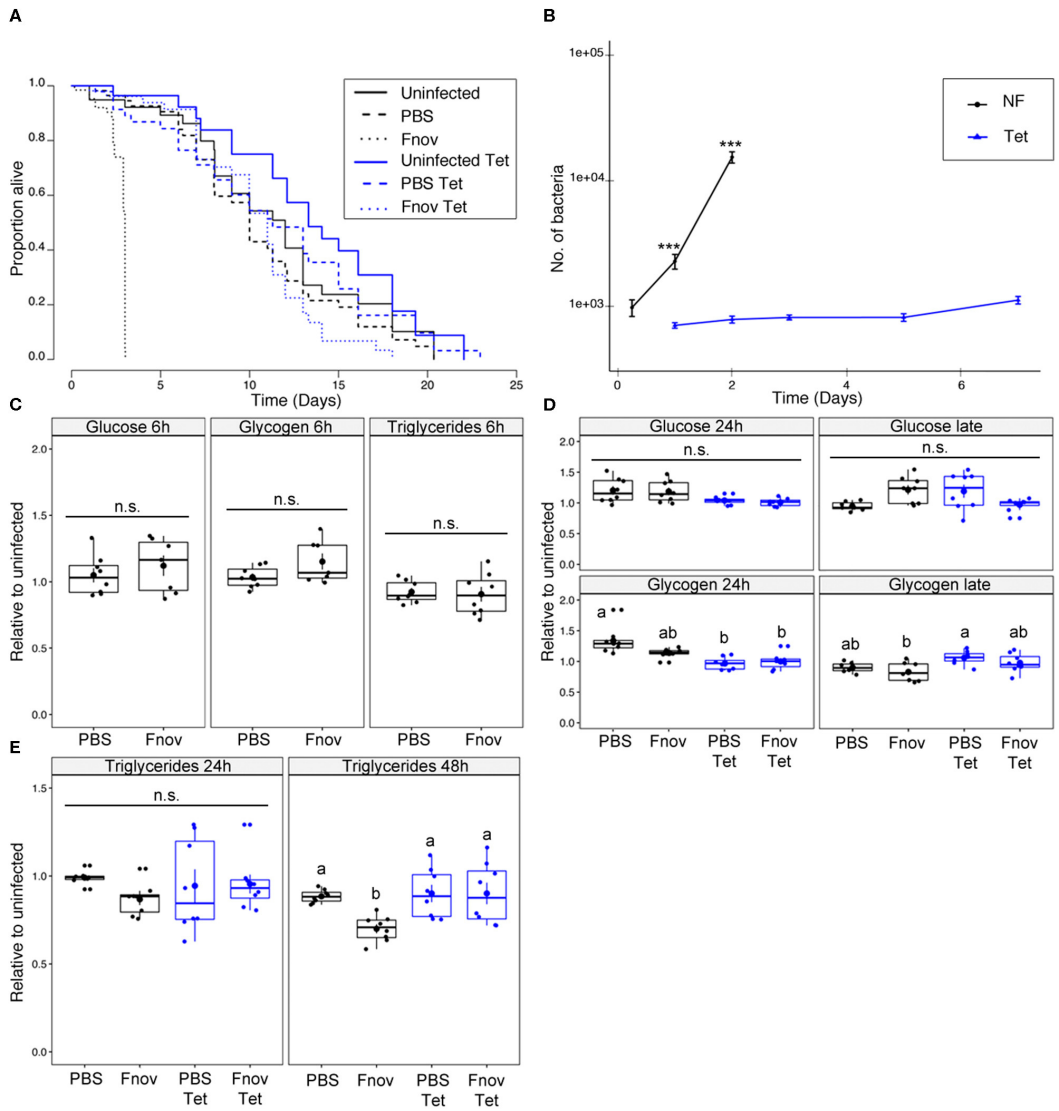
*imd*^101091^ mutants only partly reproduce metabolic phenotype of *F. novicida* infection. Five to nine days old adult *imd*^10191^ flies infected with *F. novicida* (OD_600_ = 0.1, or ~1,000 bacteria). Animals fed tetracycline were switched to tetracycline food 6 h post-infection. In all plots, black and blue tracings represent normal and tetracycline food, respectively. Significance codes: *** <0.001. Survival **(A)** *F. novicida* -infection is represented by dotted lines. Uninfected and PBS controls are represented by solid and dashed lines, respectively. Infected, tetracycline-fed flies lived 3.6x longer than flies kept on normal food; median survival of infected flies: tetracycline−11 d; normal food−3d (Log-Rank = 314.7, df = 5, *n* = 602, p ≤ 2e-16). Survivals were repeated 2 or 3 times with 20 flies/treatment/repeat. A full-factorial report of statistics can be found in Supplementary Table 2. Bacterial quantification **(B)** tetracycline-fed flies (Tet) have significantly lower bacterial loads both 1 and 2 d post infection (Kruskal-Wallis = 105.03, df = 7, *p* = 2.2e-16; Dunn's *post hoc*: 24 h–*p* = 1.3e-06, 48 h–*p* = 9.2e-11). Normal food (NF) flies show an exponential increase in bacterial load throughout infection. Load in tetracycline-fed flies remained constant throughout infection. Markers indicate means and bars represent SE. Bacterial quantifications were repeated twice, *n* = 8 samples/treatment/repeat. Metabolism **(C)** glucose, glycogen and triglyceride levels at an early point (6 h post-infection) of infection. **(D)** Glucose and glycogen levels “late” in infection (48 and 72 h for normal food and tetracycline-fed flies, respectively). Neither infection nor tetracycline affected glucose or glycogen levels. Groups sharing the same letter are not significantly different. **(E)** Infection led to a significant depletion of triglycerides during late infection in normal food flies (AOV: df =3, *n* = 28, *F* = 5.48, *p* = 4.3e-02, Tukey's HSD: normal food PBS—normal food Fnov, *p* = 0.021). Infection had no effect on tetracycline-fed flies. Large circular markers indicate means while smaller circles represent individual data points. Horizontal bar within each box represents the median. The bottom and top lines of the box represent the 1st and 3rd quartiles, respectively. Whiskers represent either the maximum and minimum values, or, the maximum and minimum values falling within 1.5x the interquartile range, in which case outliers are indicated. Groups sharing the same letter are not significantly different. Metabolic assays were repeated twice, with four samples/treatment/repeat.

Infected *imd*^10191^ flies given tetracycline lived 3.6x longer than flies kept on normal food (median survival of infected flies: tetracycline−11 days; normal food−3 days; Figure 2A, Supplementary Table 2). In the absence of infection, tetracycline had a slightly positive, though non-significant effect on survival, with uninfected normal food and tetracycline flies having similar median survival times (12 and 13.3 d, respectively).

In contrast to our observation in *w*^1118^, tetracycline did not affect survival of PBS-injected *imd*^10191^ mutants. Bacterial load differed significantly between tetracycline and normal food *imd*^10191^ flies for all time points measured (Figure 2B). Bacterial load in normal food flies differed significantly between all consecutive time points, but remained constant in tetracycline-fed flies, excluding day 7, where values were significantly higher (Figure 2B). As both survival and bacterial proliferation in *imd*^10191^ mutants resembled the overall pattern observed in *w*^1118^ flies, we tested whether the metabolic dysregulation was also present during this infection. Infection affected neither glucose nor glycogen, independent of whether flies were fed tetracycline (Figures 2C,D). Similar to what we observed in *w*^1118^, during late-stage infection, flies kept on normal food had significant depletion of triglycerides compared to PBS controls. Triglyceride levels were unaffected by infection in tetracycline-fed flies (Figure 2E).

### Pathology of Infection With Tetracycline-Resistant *F. novicida*

**Figure 3:**
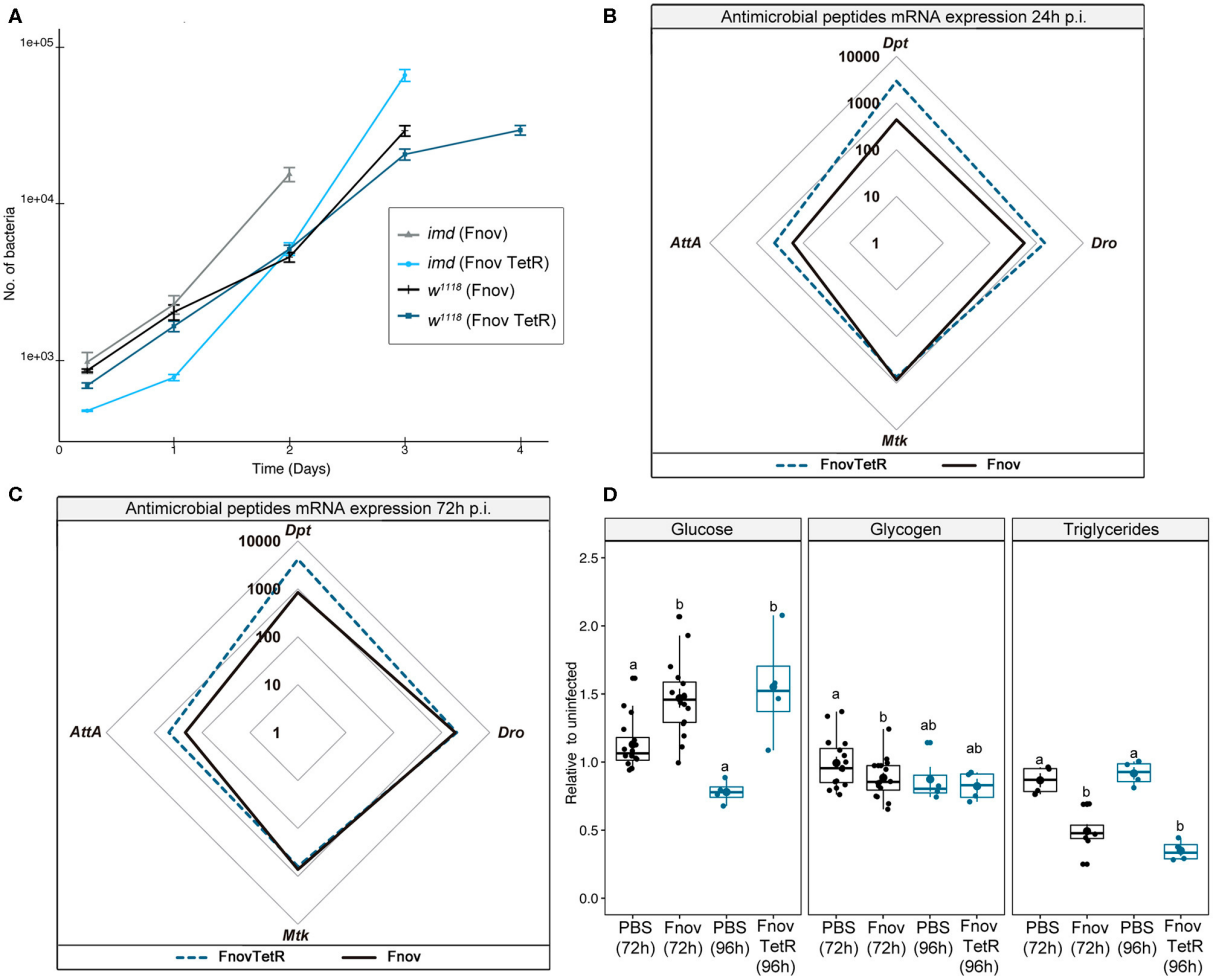
Tetracycline alone is not protective. Five to nine days old adult *w*^1118^ flies infected with tetracycline resistant *F. novicida* (TetR: OD_600_ = 0.1). Tet-R-infected animals were put on tetracycline food at the time of injection. Bacterial quantification **(A)** TetR is indicated in dark blue and light blue, and wild-type *F. novicida* in black and gray, in *w*^1118^ and *imd*^10191^ flies, respectively. TetR proliferate at a similar rate to wild-type *F. novicida*. Circular markers indicate means and bars represent SE. Bacterial quantifications were repeated twice, *n* = 8 samples/treatment/repeat. Spider plots showing antimicrobial peptide transcript levels 24 h **(B)** and 72 h **(C)** post- infection. All tracings are relative to uninfected individuals of the same treatment. TetR and wild-type *F. novicida* are indicated in dark blue dashed and solid black lines, respectively. The area contained within the innermost quadrilateral represents induction levels falling between one and ten times that of the uninfected controls. The middle and outer quadrilaterals represent 10–100 and 100–1,000-fold induction, respectively. Antimicrobial peptide assays were repeated twice, with four samples/treatment/repeat. These data are also shown, represented differently, in Supplementary Figure 3B. Metabolism **(D)** late-stage infection (normal food−72 h; tetracycline−96 h) glucose, glycogen and triglyceride levels. TetR and wild-type *F. novicida* are indicated in dark blue and black, respectively. Infection with both wild-type and TetR *F. novicida* led to hyperglycaemia and depleted triglycerides, relative to their PBS controls (Glucose: Kruskal-Wallis = 11.98, df = 3, *p* = 1.4e-5; Dunn's *post hoc*: TetR—*p* = 5.3e-04, wild-type—*p* = 2.8e-3; Triglycerides: Kruskal-Wallis = 24.66, df = 3, *p* = 3.06e-6; Dunn's *post hoc*: TetR—*p* = 2.1e-05, wild-type—*p* = 4.6e-4). Glycogen levels were unaffected in the overall model (Kruskal-Wallis = 7.171, df = 3, *p* = 6.6e-2), but analysis of only wild-type *F. novicida*- infected flies showed reduced glycogen relative to PBS controls (Wilcoxon = 164.5, *p* = 3.2e-2) whilst TetR *F. novicida* did not (Wilcoxon = 7, *p* = 0.8). Overall, we found no difference across metabolism between the two *F. novicida* strains. Large circular markers indicate means while smaller circles represent individual data points. Horizontal bar within each box represents the median. The bottom and top lines of the box represent the 1st and 3rd quartiles, respectively. Whiskers represent either the maximum and minimum values, or, the maximum and minimum values falling within 1.5x the interquartile range, in which case outliers are indicated. Groups sharing the same letter are not significantly different. Metabolic assays were repeated 2 or 3 times, with four samples/treatment/repeat.

Flies kept on tetracycline food post-injection showed restricted bacterial growth and failed to exhibit metabolic dysregulation over the course of infection (Supplementary Figure 6). To confirm that the absence of metabolic pathology in tetracycline-fed flies was the result of low pathogen loads, rather than tetracycline having a protective effect on host physiology, we repeated all experiments with tetracycline-resistant *F. novicida* (U112 pKK219—GFP, herein referred to as TetR *F. novicida*). Flies infected with TetR *F. novicida* have a median survival of 5 d (Supplementary Figure 7A), 1 day longer than infection with wild-type *F. novicida*. We attribute this difference in survival to the decreased growth rate that typically accompanies antibiotic resistance in bacteria [Supplementary Figure 7B (34, 35)], despite this difference being marginal at all timepoints assayed *in vivo* (Figure 3A). There was no difference in survival between normal food and tetracycline-fed flies infected with TetR *F. novicida*, confirming that the extended lifespan observed in TetR-infected flies (fed tetracycline) is not the result of tetracycline having an unidentified effect on the flies (Supplementary Figure 7A).

Tetracycline-resistant *F. novicida* induced AMP expression to levels equal to or greater than that of wild-type bacteria (Figures 3B,C). This confirmed that tetracycline alone did not inhibit AMP induction. To ensure that we were looking at a comparable stage of infection, we assayed metabolism at 96 h (24 h prior to death) during *TetR F. novicida* infection, rather than 72 h as with the wild-type *F. novicida*. Neither glucose nor glycogen levels differed between *TetR F. novicida* and wild-type *F. novicida*—infected flies (Figure 3D). TetR *F. novicida*—infected flies exhibited metabolic dysregulation mirroring that of wild-type *F. novicida* infections.

**Figure 4:**
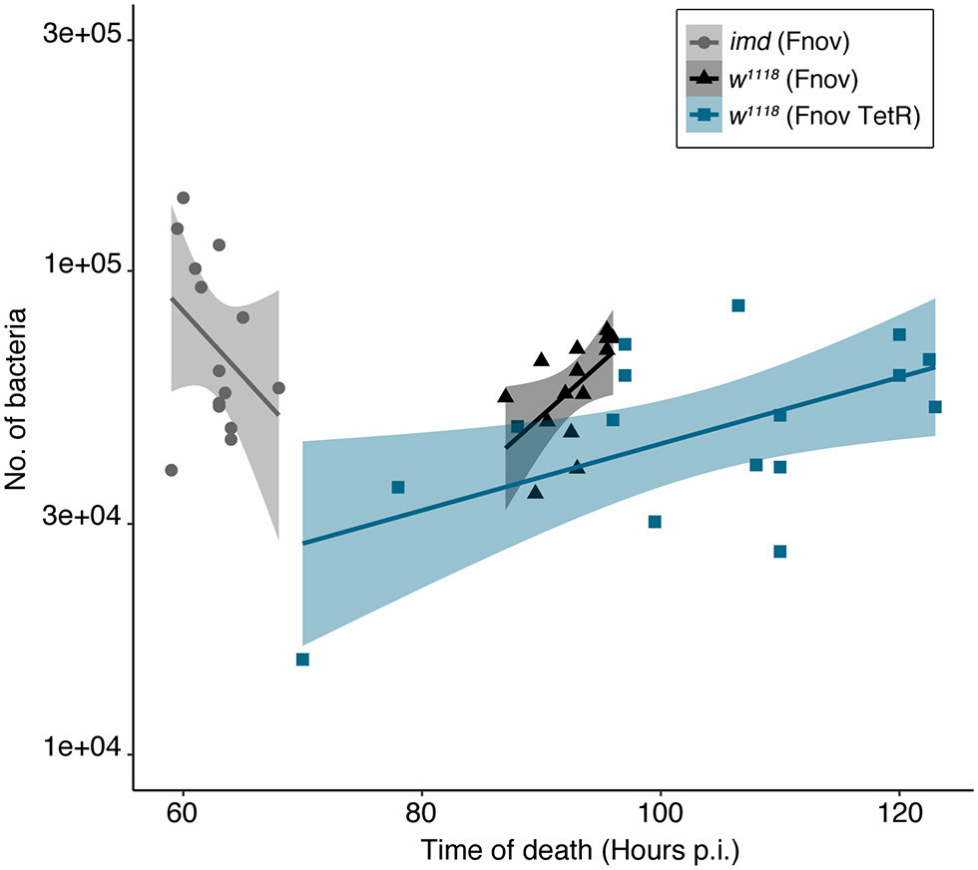
Bacterial load upon death is consistent within and across infections. Bacterial load in flies was measured within 30 min of death. We found that BLUD did not differ significantly across treatments (Kruskal-Wallis = 5.492, df = 2, *p* = 0.064). Time of host death did not correlate with bacterial load in any of the infections assayed (Pearson's correlation: *F. novicida*: *imd*^10191^—*t* = −1.677, df = 12, *p* = 0.119, *w*^1118^—*t* = 0.86363, df = 13, *p* = 0.403; TetR *F. novicida*: *w*^1118^—*t* = 1.5432, df = 15, *p* = 0.1436). *imd*^10191^ flies had the highest pathogen burden, harboring on average, 37 and 59% more bacteria than *w*^1118^ flies infected with wild-type and TetR *F. novicida*, respectively (*F. novicida*: *imd*^10191^—75 938 ± 8757, *w*^1118^—55 317 ± 4622; TetR *F. novicida* 47 505 ± 5150; mean ±se). *Francisella novicida—*infected *imd*^10191^ and *w*^1118^ flies are indicated in gray and black, respectively. TetR *F. novicida—*infected *w*^1118^ flies are indicated in blue. Each marker represents an individual fly. Standard error is demarcated by shaded area. BLUD assay was repeated twice, with six to eight samples/treatment/repeat.

We measured bacterial load upon death (BLUD) to determine whether the increased severity of metabolic dysregulation we observe over the course of infection is dependent on bacterial number. We found that BLUD did not differ significantly across treatments (Figure 4). Despite exhibiting the least amount of metabolic dysregulation, at their time of death, *imd*^10191^ flies exhibited the highest pathogen burden, harboring on average, 37 and 59% more bacteria than *w*^1118^ flies infected with wild-type and TetR *F. novicida*, respectively (*F. novicida*: *imd*^10191^—75 938 ± 8757, *w*^1118^—55 317 ± 4622; TetR *F. novicida* 47 505 ± 5150; mean ± se). We did not observe a significant correlation between time of death and bacterial load in any infection (Figure 4).

## Discussion

We have shown that infection with *F. novicida* leads to metabolic dysregulation in *w*^1118^ flies and that part of this pathology results from a direct interaction between host and pathogen, as pathology increased concomitant with bacterial load. However, activation of the *imd*-derived antimicrobial peptide response also contributes to this phenotype as we found both that: *imd* mutants do not exhibit similar levels of dysregulation, and; *w*^1118^ flies treated with tetracycline exhibited both a strong induction of AMPs, as well as a trend toward hyperglycaemia and triglyceride depletion (Figure 1). Whilst these subtle trends found in tetracycline-treated flies could be attributed to low bacterial loads (rather than immune activation), hyperglycaemia and triglyceride depletion were not seen in tetracycline-treated *imd* mutants, demonstrating that immune costs—rather than bacterial load—were responsible. However, that we do see triglyceride depletion in normal-food *imd* flies, suggests that at least part of this phenotype is pathogen-driven, possibly resulting from host triglyceride usurpation by the bacteria (36–38).

It is still possible that the metabolic pathology we observe is a consequence of *Toll* pathway activation, notwithstanding the fact that most immune activation in this infection is *imd*-dependent. Others have observed that Gram-negative infections can activate the *Toll* pathway (9, 39–41), and Toll pathway activation has been associated with effects on the insulin signaling pathway that could drive the metabolic effects we see in this infection. This possibility—that bacterial sensing via the *Toll* pathway is the critical driver of metabolic pathology in most bacterial infections in *Drosophila*, independent of the specific type of bacterium—is an interesting one for future study.

One further possibility is that *F. novicida* makes direct use of host-derived resources and therefore is responsible for some of the changes in host metabolism we observe. *Francisella* spp. preferentially use glucose as a carbon source (38, 42). It has also been shown that *F. tularensis* cannot use glycogen as a carbon source (43); thus, if glycogen depletion were the result of bacterial consumption of nutrients, the expected pathway would be via bacterial depletion of free sugar triggering host glycogenolysis. We observe that glycogen depletion is accompanied by increases in free sugar, such immunometabolic switches are well-known (44) and have been shown to be instrumental in the provisioning of resources to haemocytes during infection in *D. melanogaster* (45), further suggesting that metabolic demands of the host—rather than pathogen—account for much of the observed effect.

During intracellular replication, *F. novicida* has been shown to metabolize glycerol for gluconeogenesis (38), supporting the possibility that the observed triglyceride depletion was bacterial-driven. However, in *D. melanogaster*, proliferation of *F. novicida* occurs predominantly extracellularly (22), and it is unclear how large of an impact these bacteria could have on host triglyceride levels. None of this precludes the possibility that *F. novicida* triggers host catabolism of endogenous stores to convert them into a form it can use itself. However, the fact that hyperglycaemia and loss of triglyceride and glycogen stores is seen in other bacterial infections, does imply that this reflects an aspect of the host response (20, 46), and the presence of hyperglycaemia in this infection suggests that the amount of sugar being released exceeds the amount consumed by the bacteria.

Finally, the metabolic pathology observed may be part of a moribund phenotype, with an overall worsening of condition as the animal approaches death. However, if this were the case, we would not expect to observe said pathology during infection with non-lethal bacteria like *M. luteus* (Supplementary Figure 4). Furthermore, if metabolic dysregulation and bacterial load are decoupled, as should be the case if it is caused by moribundity, we would have expected tetracycline-fed flies to exhibit metabolic dysregulation as this infection persists for several days and activates the immune response to levels comparable to flies fed on normal food.

Together these data indicate that in this infection *imd* activation is necessary, but not sufficient, for metabolic pathology. Tetracycline-fed flies of both genotypes maintain constant metabolite levels, and *w*^1118^ maintain AMP levels, throughout the duration of the infection (Supplementary Figure 6); this may be due in part, to an effect of tetracycline on host physiology and metabolism (47). We found no difference in triglyceride levels between uninfected, normal food flies of the two genotypes (Supplementary Figure 7); thus, the differential usage of energy stores between *w*^1118^ and *imd*^10191^, is unlikely the result of disparate resource availability. Previous work in our lab suggests that during bacterial infection, flies may disrupt insulin signaling as a means of conserving energy for immune-related activities, leading to metabolic dysregulation (20). In showing that in the absence of *imd*, part of the metabolic phenotype observed during *w*^1118^ infection cannot be reproduced, the current work supports this supposition. Additionally, as *imd* mutants retain an active Toll response, this also demonstrates that Toll is not—at least entirely—responsible for the observed phenotype.

Flies infected with TetR *F. novicida* and kept on tetracycline-food exhibited near-identical infection pathology—albeit slightly protracted—to wild-type *F. novicida* infection. Interestingly, we found that independent of host and pathogen identity and their interaction, flies had similar pathogen loads at the time of death, despite there being a 1–2 d difference in median survivals (Figure 4); this observation further supports the idea that there is a critical bacterial load beyond which hosts cannot survive (12).

The metabolic dysregulation observed during infection is likely the result of several different factors. That multiple bacteria cause some sort of metabolic shift in their hosts suggests that both bacterial and host factors contribute to the phenotype; elucidating the need to understand the different requirements of pathogens and how these are met in a given host. Further investigation into the cross-talk between host immune and metabolic pathways under different infections has the potential to reveal some of this interaction.

## Data Availability Statement

All datasets generated for this study are included in the article/Supplementary Material.

## Author Contributions

CV conceived, designed and performed experiments, analyzed data, and wrote the manuscript. CS performed experiments, prepared figures, and edited the manuscript. AW performed experiments and edited the manuscript. MD conceived of experiments and edited the manuscript. All authors contributed to the article and approved the submitted version.

## Conflict of Interest

The authors declare that the research was conducted in the absence of any commercial or financial relationships that could be construed as a potential conflict of interest.
