# Supplementary material for: Origins of Metabolic Pathology in Francisella-Infected Drosophila
Source: Front Immunol. 2020 Jul 8;11:1419. doi: 10.3389/fimmu.2020.01419 (PMC7360822; doi:10.3389/fimmu.2020.01419)
Supplement: Supplementary file 1 [file Data_Sheet_1.PDF]

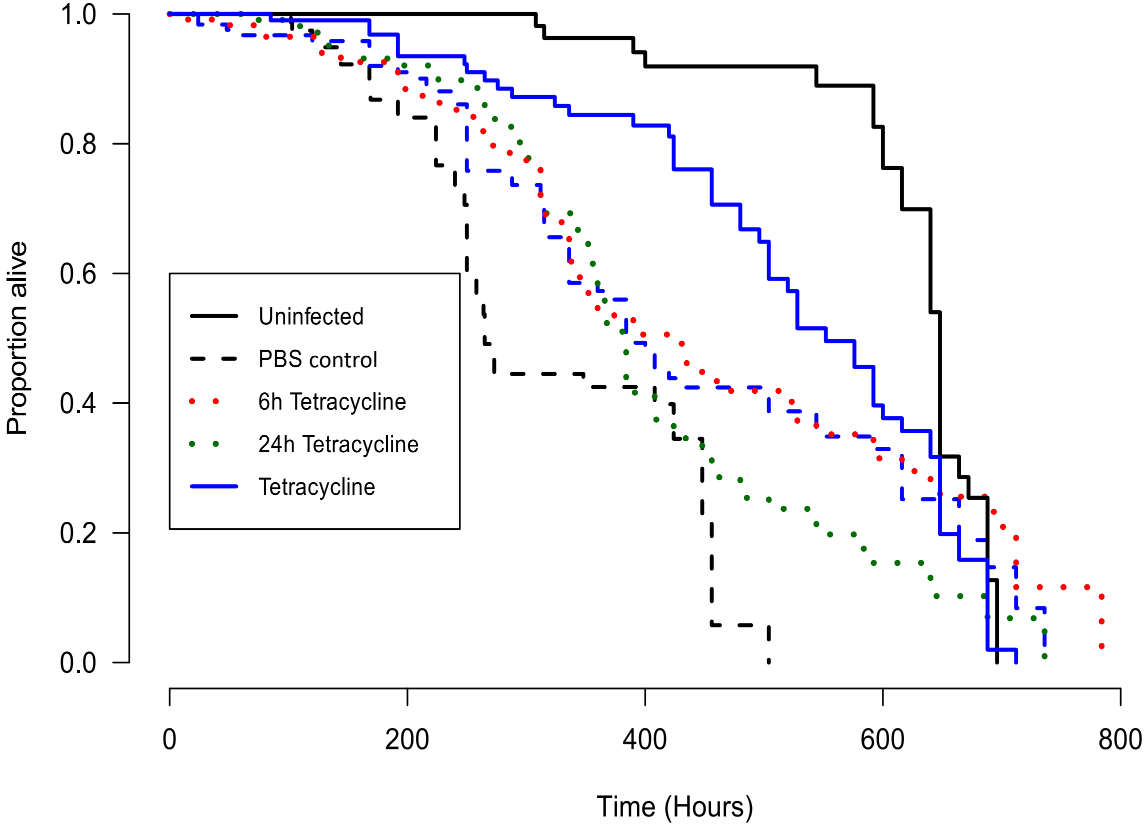

SI Fig 1 . 5 –9d old adult *w*^{1118} flies infected with *F. novicida* (OD600 = 0.1, or ~1,000 bacteria). Infected animals were switched to tetracycline food either 6h (orange–dotted line) or 24h (green -dotted line) post infection. Black and blue tracings represent normal and tetracycline food, respectively. Solid and dashed lines indicate uninfected and PBS controls, respectively. Median survival of tetracycline-fed infected flies: 6h -17.5d; 24h –16d. Survival was repeated thrice with 20 flies/treatment.
