# Supplementary material for: Origins of Metabolic Pathology in Francisella-Infected Drosophila
Source: Front Immunol. 2020 Jul 8;11:1419. doi: 10.3389/fimmu.2020.01419 (PMC7360822; doi:10.3389/fimmu.2020.01419)
Supplement: Supplementary file 2 [file Data_Sheet_2.PDF]

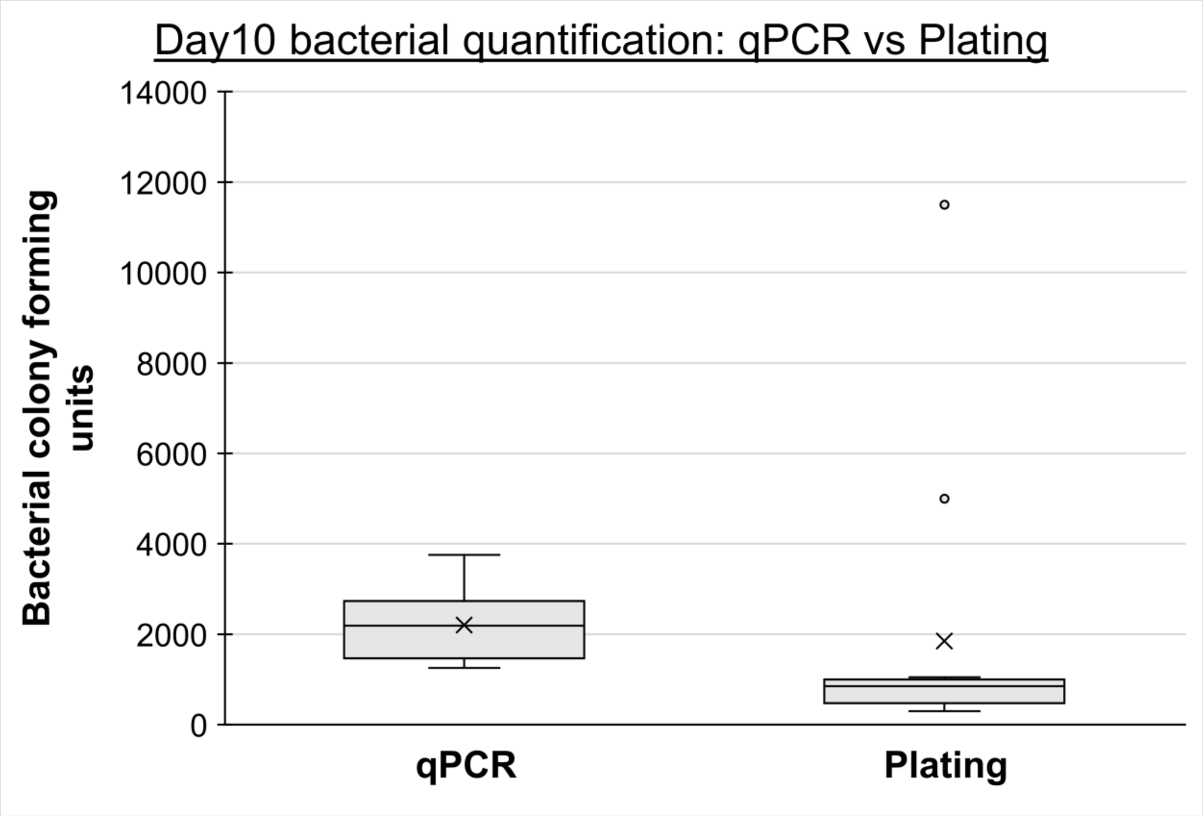

SI Fig 2. Comparison of plating and qPCR methods for quantifying bacterial number. 5 – 9d old adult w^{1118} flies infected with *F. novicida* (OD600 = 0.1, or ~1,000 bacteria). Six hours following injection animals were transferred to tetracycline food. After ten days flies were homogenised and number of bacteria quantified either via qPCR (as described in methods, n = 16) or plating (n = 13). When plating, for each sample, 1 fly was homogenised in 100µl of sterile ddH_2O. Homogenates were serially diluted and plated onto TSB agar plates where they incubated for 16-24h. Following incubation, the number of individual bacterial colonies observed on each plate was quantified and back-calculated to determine the number of CFUs present in each fly. We observed a 38 – fold and 2.2 – fold difference within plating and qPCR data respectively; given this, we chose to quantify bacteria via qPCR as its level of variation is biologically explicable, suggesting that this method is less susceptible to experimental error. 'X' indicates average and the horizontal bar within each box represents the median. The bottom and top lines of the box represents the 1st and 3rd quartiles, respectively.
