# Supplementary material for: Origins of Metabolic Pathology in Francisella-Infected Drosophila
Source: Front Immunol. 2020 Jul 8;11:1419. doi: 10.3389/fimmu.2020.01419 (PMC7360822; doi:10.3389/fimmu.2020.01419)
Supplement: Supplementary file 3 [file Data_Sheet_3.PDF]

A

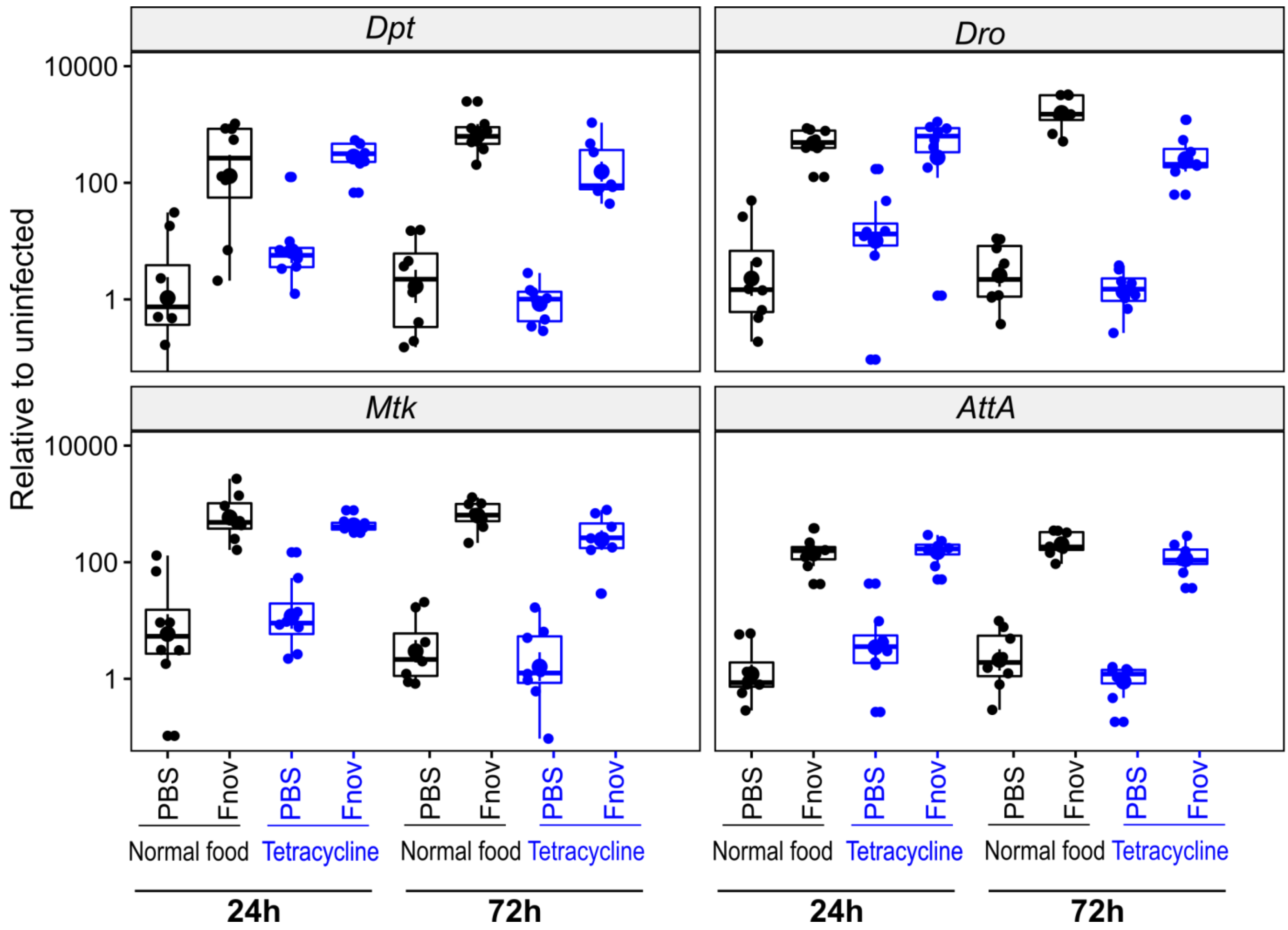

B

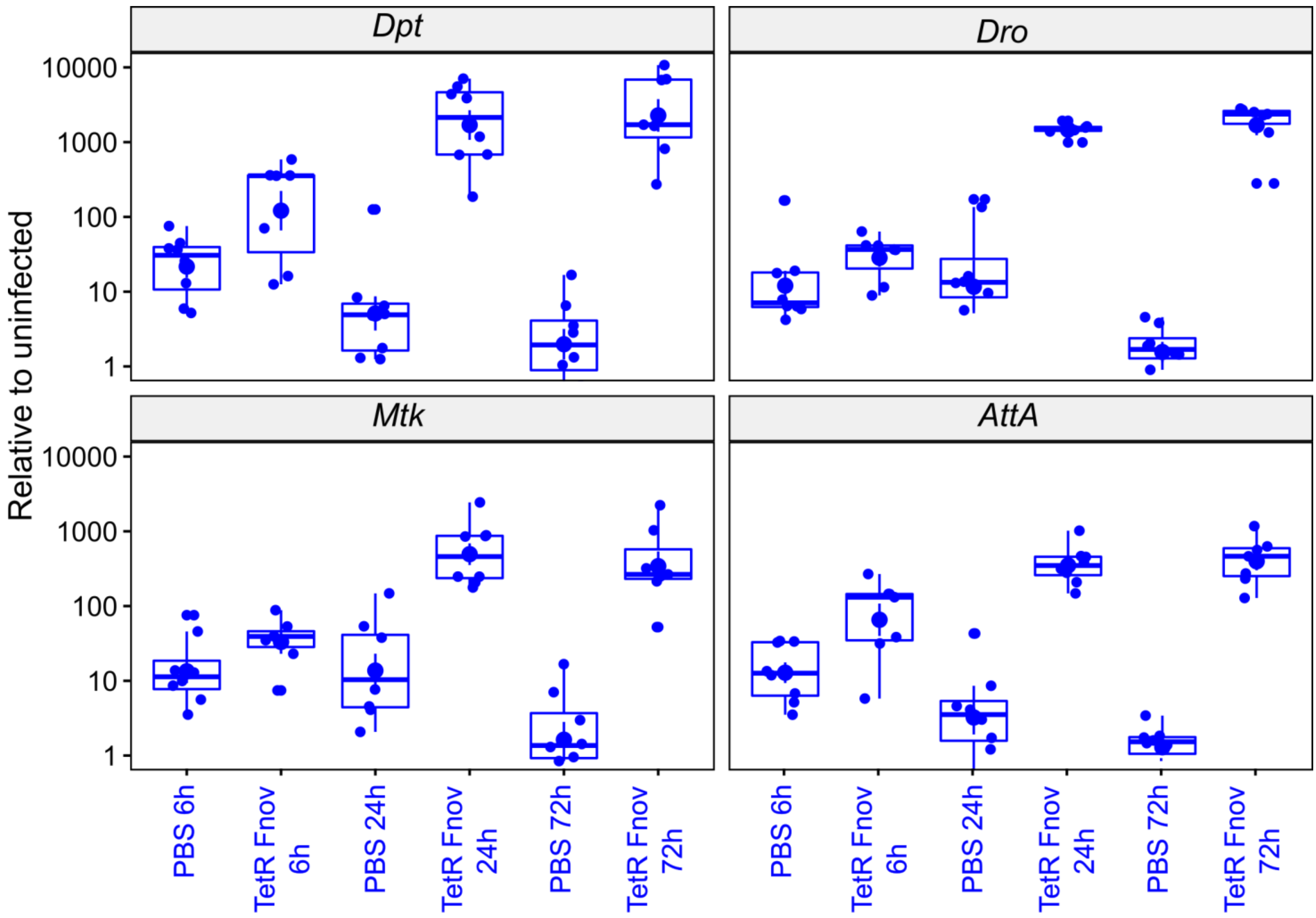

SI Fig 3. Boxplots depicting AMP expression (mRNA) in flies infected with **(A)** wild-type, and **(B)** TetR *F. novicida*). Treatments where flies were fed tetracycline are indicated in blue. Large circular markers indicate means while smaller circles represent individual data points. Horizontal bar within each box represents the median. The bottom and top lines of the box represent the 1st and 3rd quartiles, respectively. Whiskers represent either the maximum and minimum values, or, the maximum and minimum values falling within 1.5x the interquartile range, in which case outliers are indicated.
