# Supplementary material for: Origins of Metabolic Pathology in Francisella-Infected Drosophila
Source: Front Immunol. 2020 Jul 8;11:1419. doi: 10.3389/fimmu.2020.01419 (PMC7360822; doi:10.3389/fimmu.2020.01419)
Supplement: Supplementary file 6 [file Data_Sheet_6.PDF]

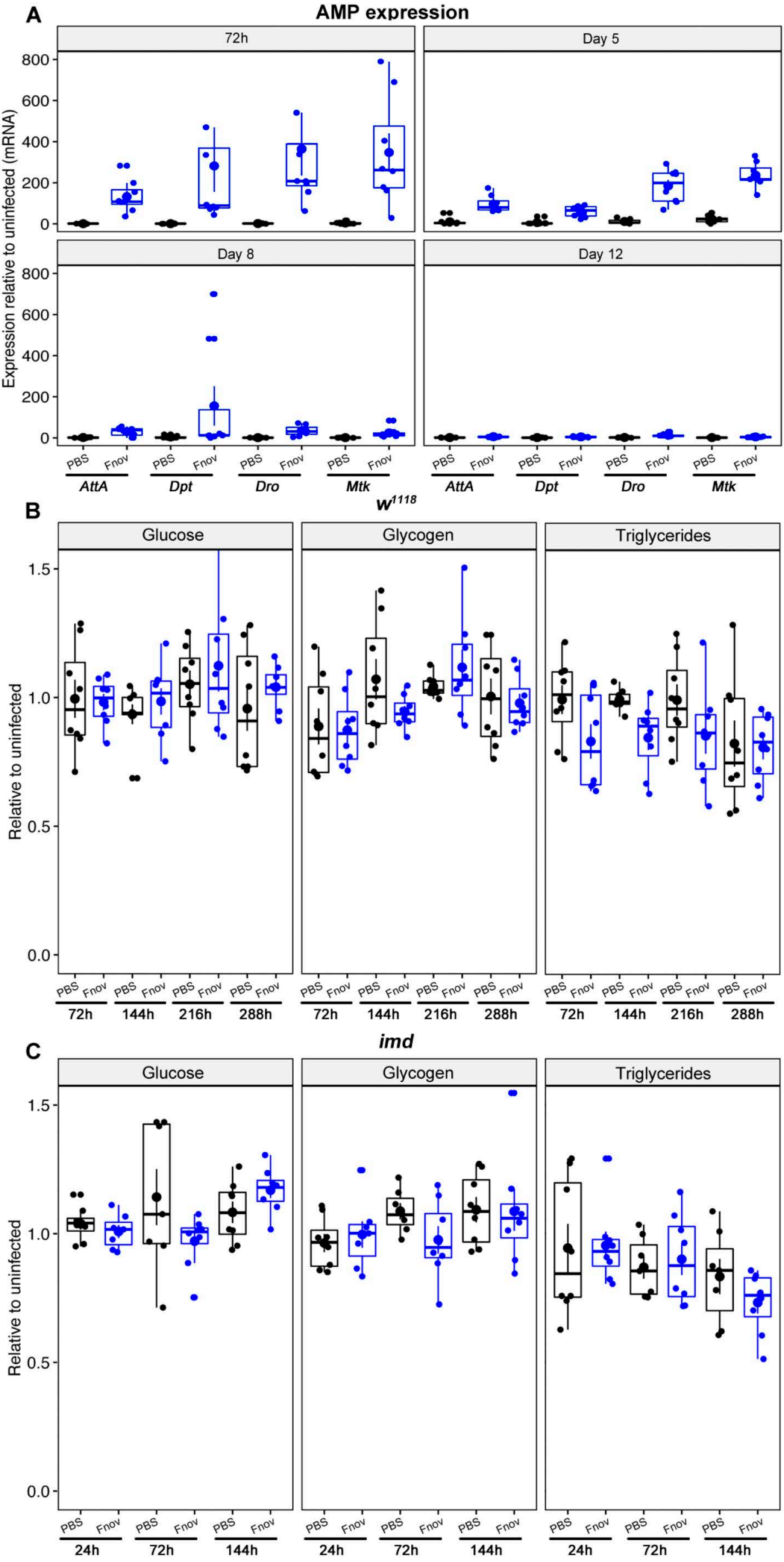
A
AMP expression
72h
Day 5
Day 8
Day 12
Expression relative to uninfected (mRNA)
PBS
Fnov
AttA
Dpt
Dro
Mtk
B
w1118
Glucose
Glycogen
Triglycerides
Relative to uninfected
72h
144h
216h
288h
C
imd
24h
72h
144h

SI Fig 6. Late-infection AMPs and metabolism of tetracycline-fed flies. 5 – 9d old adult flies infected with *F. novicida* (OD600 = 0.1, or ~1,000 bacteria). Six hours following injection animals were transferred to tetracycline food. **(a)** AMP expression of w^{1118} flies infected with *F. novicida* and given tetracycline. Expression levels are relative to uninfected controls. Black and blue tracings indicate PBS controls and *F. novicida* infected flies, respectively. w^{1118} **(b)** and imd^{10191} **(c)** glucose, glycogen and triglyceride levels are shown, relative to uninfected controls. Black and blue tracings indicate PBS controls *F. novicida* infected flies, respectively. For all plots, large circular markers indicate means while smaller circles represent individual data points. Horizontal bar within each box represents the median. The bottom and top lines of the box represent the 1st and 3rd quartiles, respectively. Whiskers represent either the maximum and minimum values, or, the maximum and minimum values falling within 1.5x the interquartile range, in which case outliers are indicated. Experiments were repeated 2 or 3 times.
