# Supplementary material for: Origins of Metabolic Pathology in Francisella-Infected Drosophila
Source: Front Immunol. 2020 Jul 8;11:1419. doi: 10.3389/fimmu.2020.01419 (PMC7360822; doi:10.3389/fimmu.2020.01419)
Supplement: Supplementary file 7 [file Data_Sheet_7.PDF]

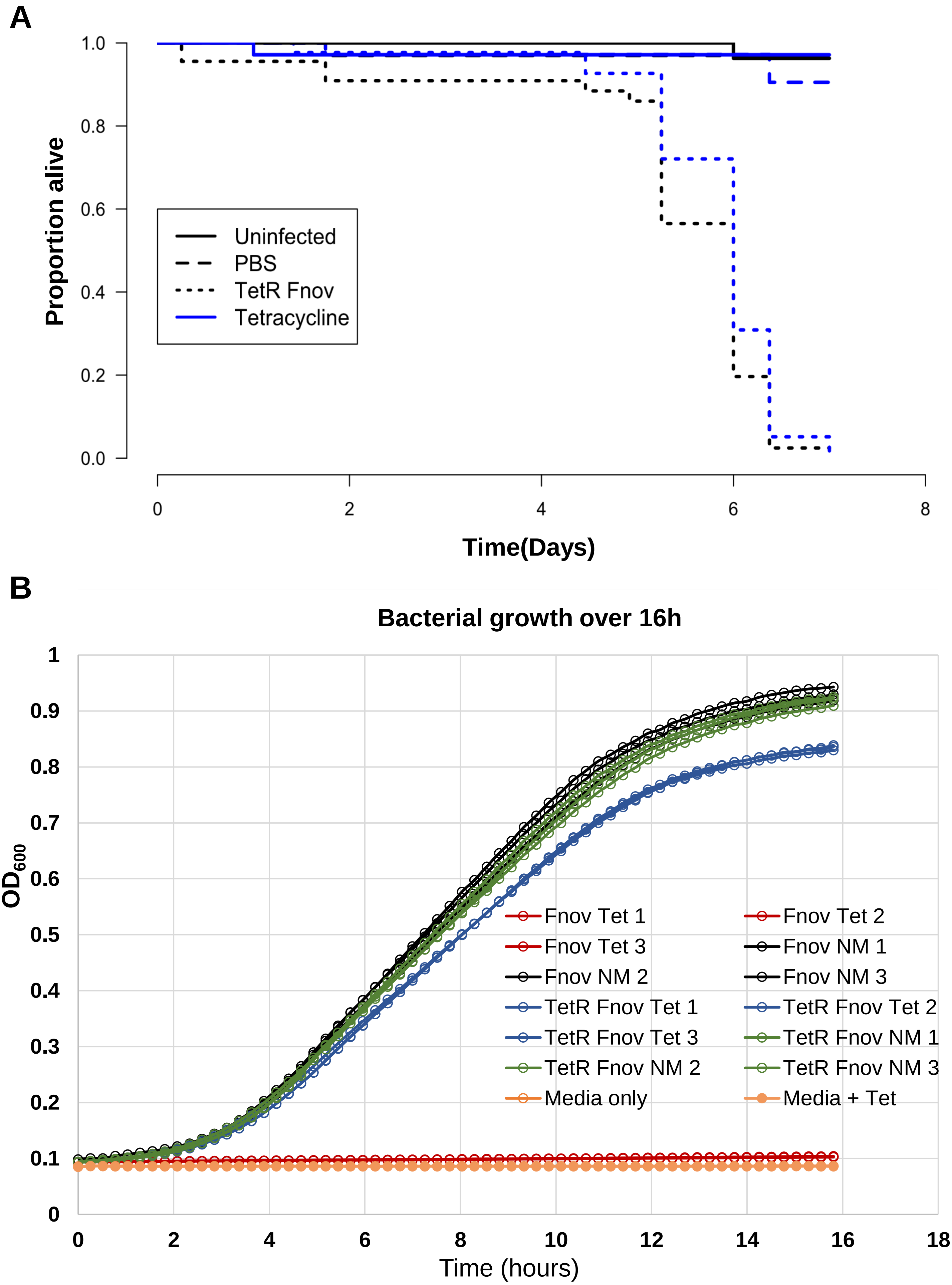

SI Fig 7. Kinetics of wild-type and tetracycline resistant *F. novicida*. (A) 5 – 9d old adult w^{1118} flies infected with TetR *F. novicida*. Animals fed tetracycline were put on tetracycline food immediately following injection. TetR *F. novicida* -infection is represented by dotted lines. Uninfected and PBS controls are represented by solid and dashed lines, respectively. Median time to death did not differ between flies on and off tetracycline food (144h). Black and blue tracings represent normal and tetracycline food, respectively. Survival was repeated twice with 20 flies/treatment/repeat. **(B)** Growth curves of *F. novicida*. Wild-type *F. novicida* grown in normal media (NM) is indicated in black. As expected, the wild-type strain did not grow in media supplemented with tetracycline (red tracings). TetR *F. novicida*, indicated in blue, have impaired growth in the presence of tetracycline. In the absence of tetracycline (green tracing), TetR bacteria grow at a rate similar to that of wild-type. Data represent 3 biological replicates as indicated (“1”, “2”, “3”). The average of 4 technical replicates of each biological replicate is shown. No growth was observed in media only cultures or media + tetracycline cultures. Growth assay was repeated twice; results of one replicate are shown and are representative of the second.
