# Supplementary material for: Origins of Metabolic Pathology in Francisella-Infected Drosophila
Source: Front Immunol. 2020 Jul 8;11:1419. doi: 10.3389/fimmu.2020.01419 (PMC7360822; doi:10.3389/fimmu.2020.01419)
Supplement: Supplementary file 8 [file Data_Sheet_8.PDF]

SI Table 1. Statistical significance of pairwise comparisions (*w*)

	Uninfected	PBS	Fnov	Uninfected	PBS	Fnov	Median survival (days)
Uninfected							27
PBS	***						11
Fnov	***	***					4
Uninfected	**	***	***				23
PBS	n.s.	***	***	n.s.			16
Fnov	**	***	***	n.s.	n.s.		17.5

Tetracycline in blue　n.s. - non-significant　* - 0.05>　** - 0.01>　*** - 0.001>

SI Table 2. Statistical significance of pairwise comparisions (*imd*)

	Uninfected	PBS	Fnov	Uninfected	PBS	Fnov	Median survival (days)
Uninfected							12
PBS	n.s.						10
Fnov	***	***					3
Uninfected	n.s.	*	***				13.3
PBS	n.s.	n.s.	***	n.s.			11.3
Fnov	n.s.	n.s.	***	**	n.s		11

Tetracycline in blue　n.s. - non-significant　* - 0.05>　** - 0.01>　*** - 0.001>
